# Protecting Kidney Health in the First 1000 Days: The Pediatrician’s Role in Safeguarding the Weakest

**DOI:** 10.3390/diseases14050151

**Published:** 2026-04-22

**Authors:** Luca Pecoraro, Ilenia Chillura, Agnese Bigioni, Maria Maddalena Quarta, Emiliano Altavilla, Enrico Rosati, Flavia Indrio

**Affiliations:** 1 Department of Experimental Medicine, Pediatric Section, University of Salento Hospital “Vito Fazzi”, 73100 Lecce, Italy; 2 Pediatric Unit, Ospedale Vito Fazzi, ASL Lecce, 73100 Lecce, Italy; 3 Pediatric Department, University of Bari Aldo Moro, 70121 Bari, Italy; 4 Neonatology and Intensive Care Unit, “Vito Fazzi” Hospital, 73100 Lecce, Italy

**Keywords:** kidney development, first 1000 days of life, congenital factors, at-risk newborns, kidney size, kidney function, early assessment, vulnerable infants

## Abstract

Kidney development in the first 1000 days of life is vulnerable to numerous prenatal, perinatal, and congenital factors. This review aims to analyze the main determinants of early kidney development and to highlight the role of pediatricians in identifying at-risk infants and implementing preventive strategies to reduce the risk of chronic kidney disease (CKD). For at-risk newborns, early assessment of kidney size and function is essential for the timely detection of functional decline. Key risk factors include prenatal exposures, perinatal complications, genetic conditions, and postnatal factors. Early, tailored nephrological follow-up is crucial for preventing CKD and its complications. Determining optimal monitoring intervals through clinical, laboratory, and ultrasound evaluations enables risk stratification, ensuring closer surveillance for the most vulnerable infants during this critical window. This review integrates evidence from experimental, epidemiological, and clinical studies and highlights the importance of early-life interventions in shaping renal health across the lifespan.

## 1. Introduction

The first 1000 days of life—from preconception through pregnancy to the end of the second year—represent a critical window for long-term organ development and overall health [1]. During this period, rapid physiological changes interact with environmental exposures to shape the structural and functional trajectories of multiple organ systems. Kidney development is particularly complex and time sensitive. Nephrogenesis begins early in gestation and continues until 34–36 weeks, with nearly two-thirds of nephrons forming during the last trimester [2]. As nephron numbers are fixed at birth and no postnatal regeneration occurs, early renal endowment is a key determinant of lifelong kidney health [2,3]. These developmental processes are highly vulnerable to disruption. Suboptimal intrauterine conditions, maternal disease, placental dysfunction, and environmental or pharmacological stressors may impair nephron formation and renal maturation [4]. Within the developmental origins of health and disease framework, early-life stressors can induce epigenetic changes that affect renal and cardiovascular development, thereby increasing susceptibility to later diseases. In addition, perinatal complications and early postnatal challenges may further compromise renal adaptation to extrauterine life [5]. Several biological mechanisms support this developmental programming model. Dysregulation of the renin–angiotensin–aldosterone system during nephrogenesis can impair nephron formation and renal vascular development, predisposing to long-term alterations in blood pressure regulation [6]. Early oxidative stress may disrupt endothelial maturation and promote microvascular rarefaction, linking adverse exposures to hypertension and chronic kidney disease. Epigenetic mechanisms, including DNA methylation, histone modifications, and microRNA regulation, can act as a molecular memory of early insults, leading to persistent changes in gene expression. Consistently, impaired renal microvasculature and endothelial dysfunction have been observed early in individuals exposed to adverse intrauterine environments. Together, these mechanisms provide a biological basis for the association between early adversity and chronic renal and cardiovascular disease. Certain neonatal populations are at increased risk of reduced nephron endowment or early renal vulnerability. Preterm infants, particularly those born before completion of nephrogenesis, may have fewer and structurally abnormal nephrons [6]. Low birth weight and intrauterine growth restriction—often related to placental insufficiency or maternal conditions such as pre-eclampsia and gestational diabetes—are associated with altered renal structure, reduced functional reserve, and increased lifetime risk of chronic disease [7,8,9]. Additional risk factors include congenital anomalies of the kidney and urinary tract, genetic syndromes, exposure to nephrotoxic medications, and conditions requiring intensive neonatal care [10,11]. These early determinants may interact with postnatal factors—such as nutrition, infections, hydration status, and further nephrotoxic exposures—amplifying the long-term burden on renal health [12]. Chronic kidney disease (CKD) is an expanding public health challenge associated with significant morbidity and mortality. Its onset is often silent, and a substantial proportion of adult CKD is now thought to originate from early developmental processes rather than solely from adult risk factors [13,14]. Epidemiological evidence indicates that individuals with impaired kidney development or early-life renal insults have a higher risk of hypertension, reduced glomerular filtration rate, and albuminuria later in life [12,15,16]. This evidence highlights the importance of understanding early determinants of renal vulnerability. However, evidence-based guidelines for renal follow-up after discharge from the neonatal intensive care unit are still lacking. Given the interplay between fetal programming, perinatal events, and long-term outcomes, the first 1000 days represent a crucial period for kidney health. Early identification of at-risk infants and a better understanding of the mechanisms linking early adversity to adult disease are essential to improve lifelong renal outcomes. While congenital kidney disorders are widely recognized as risk factors for CKD, many other early-life determinants remain under-recognized and are not routinely addressed in pediatric follow-up. This review deepens understanding of how the first 1000 days influence renal health across the lifespan. It integrates evidence from experimental, epidemiological, and clinical studies and highlights the key role of pediatricians in translating this knowledge into preventive strategies.

## 2. Material and Methods

We conducted a non-systematic narrative review of the most relevant studies addressing kidney development and risk factors affecting renal health during the first 1000 days of life. The literature search was performed using the PubMed and Cochrane Library databases, covering publications from January 1990 to November 2024. The articles were identified using a combination of Medical Subject Headings (MeSH) terms and relevant free-text keywords, including their synonyms and combinations: “kidney development”, “nephrogenesis”, “first 1000 days”, “prenatal factors”, “perinatal risk factors”, “low birth weight”, “prematurity”, “intrauterine growth restriction”, “congenital anomalies”, “pediatric kidney disease”, “chronic kidney disease”, “early life exposures”, and “renal programming”. The initial search yielded 892 records. After removal of duplicates, titles and abstracts were screened for relevance to the scope of this review. Full texts of potentially eligible articles were then assessed. Studies reporting data from experimental models, epidemiological investigations, cohort studies, case–control studies, randomized controlled trials, and relevant reviews were considered eligible for inclusion. Only articles published in English were included. Exclusion criteria comprised conference abstracts, editorials, letters, discussion papers, studies lacking full text, and animal studies not directly translatable to human physiology. The selection process involved sequential screening of titles, abstracts, and full texts. Following this process, 191 studies were included in the final analysis. The literature selection and data extraction were conducted by multiple authors (L.P., F.I., A.B., I.C., E.R., E.A., and M.M.Q.), and all included data were independently reviewed to ensure accuracy and consistency.

## 3. Prenatal Risk Factors

### 3.1. Gestational Diabetes

Gestational diabetes is associated with an increased risk of renal impairment in the offspring, both in the neonatal period and later in life [17]. Exposure to maternal hyperglycemia during fetal life can disrupt nephrogenesis, resulting in reduced nephron endowment and structural and functional renal alterations. In neonates born to mothers with poorly controlled gestational diabetes, these changes are reflected by smaller renal volumes and increased urinary biomarkers of tubular injury [17]. Hyperglycemia-induced epigenetic modifications further interfere with the regulation of genes involved in kidney development, thereby increasing the risk of long-term renal dysfunction [18]. Consistently, adult offspring of diabetic mothers show a reduced renal functional reserve compared with offspring of diabetic fathers, supporting the hypothesis of lower nephron endowment due to intrauterine exposure [19]. In addition, maternal diabetes is associated with a threefold increased risk of renal agenesis and dysgenesis [20]. Gestational diabetes is also frequently associated with high birth weight, which is a known risk factor for hypertension, type 2 diabetes, renal disease, and cardiovascular disease in later life. However, its specific impact on nephron number remains unclear [21].

### 3.2. Drugs of Abuse: Tobacco, Alcohol and Cocaine

Maternal smoking during pregnancy has been causally associated with an increased risk of acute kidney injury (AKI), renal malignancies, and possibly glomerulonephritis in the offspring, as shown by Mendelian randomization analyses based on large-scale Genome-Wide Association Study (GWAS) datasets [22]. Experimental and clinical evidence indicate a dose-dependent relationship between maternal smoking and reduced fetal kidney volume, reflecting impaired nephrogenesis [23]. Nicotine, along with the oxidative and pro-inflammatory components of cigarette smoke, is considered a major contributor to these effects. The use of e-cigarettes has increased in recent years [24]. Current evidence suggests that e-cigarettes can induce inflammation and oxidative stress independently of nicotine content, indicating that they may also exert adverse effects on fetal development [24]. Maternal alcohol exposure during pregnancy has been associated with the development of mild CKD in offspring by 30 years of age [25]. Experimental models support these findings: in ethanol-exposed rats, adult offspring show reduced nephron number and impaired renal function, likely due to disrupted ureteric branching morphogenesis [26]. In humans, prenatal alcohol exposure is also linked to an increased risk of congenital renal anomalies, including renal agenesis and hypoplasia, particularly at moderate or high levels of intake [27]. Cocaine readily crosses the placenta and induces marked vasoconstriction in fetal vessels, leading to reduced placental blood flow and impaired nutrient delivery [28,29]. These effects may compromise renal development. Morphometric analyses of fetal kidneys exposed to cocaine demonstrate increased interlobular arterial wall thickness and reduced arterial luminal circumference compared with controls [30]. In addition, cocaine and other substances of abuse increase the risk of preterm birth, low birth weight, and intrauterine growth restriction, all of which are established risk factors for later renal impairment [31,32,33].

### 3.3. Maternal Infections

Urinary tract infections (UTIs) are the most common bacterial infections during pregnancy, affecting up to 15% of pregnant individuals [34]. Both asymptomatic bacteriuria and symptomatic UTIs are associated with an increased risk of preterm birth [35,36]. This association is largely mediated by bacterial endotoxins and pro-inflammatory cytokines, which stimulate prostaglandin synthesis and promote uterine contractility [37]. Chorioamnionitis represents a major cause of preterm birth and is characterized by a pronounced inflammatory response. This inflammatory milieu may interfere with normal nephrogenesis, particularly by disrupting vascular endothelial growth factor (VEGF) pathways and the renin–angiotensin–aldosterone system (RAAS) [38,39,40]. Both VEGF and RAAS are essential for glomerular and peritubular vasculogenesis during kidney development [41,42]. Disruption of these pathways during critical developmental windows may lead to persistent structural and functional renal alterations, increasing susceptibility to podocyte injury and proteinuria later in life [43].

### 3.4. Nutritional Deficiencies: Iron and Zinc

Maternal iron deficiency is associated with gestational anemia, reduced placental oxygen transport, and impaired fetal growth, leading to outcomes such as low birth weight, fetal compromise, and preterm delivery [44]. Experimental studies in rodent models show that gestational iron deficiency can also disrupt renal development, delaying nephrogenesis and resulting in a persistent reduction in nephron number in adulthood [45,46]. Zinc deficiency during prenatal and postnatal development has been shown to increase systolic blood pressure and impair renal function in adult male rats. These effects are associated with reduced nephron endowment, increased renal oxidative stress and apoptosis, and decreased activity of the renal and vascular nitric oxide system [47,48]. In addition, zinc deficiency has been linked to early structural kidney alterations and long-term changes in the renal renin–angiotensin system, potentially increasing the risk of renal and cardiovascular disease in adulthood [49].

## 4. Perinatal Risk Factors

### 4.1. Preterm Birth

Preterm birth is defined as delivery before 37 weeks of gestation [50]. Each year, approximately 15 million preterm births occur worldwide, accounting for about 10% of all births in the United States [51,52]. Although prematurity remains a major cause of infant morbidity and mortality, advances in neonatal and perinatal care have significantly improved survival. At the same time, increasing maternal age and the widespread use of assisted reproductive technologies have contributed to a higher prevalence of preterm births [53], resulting in a growing population of preterm survivors [54,55]. Preterm infants are classified according to gestational age as late (34–36 weeks), moderate (32–33 weeks), very (28–31 weeks), and extremely preterm (<28 weeks) [56]. While pulmonary and neurological outcomes are well described, the renal consequences of prematurity have only recently received greater attention [57]. Preterm infants have lower estimated glomerular filtration rate (eGFR) at birth and exhibit slower postnatal renal maturation than term newborns [58,59]. Prematurity is a well-established risk factor for CKD [60,61,62,63]. Because a substantial proportion of nephrogenesis occurs during the last trimester, preterm infants are born with a reduced nephron endowment. This deficit predisposes to proteinuria, inflammation, and tubulointerstitial fibrosis [64]. Experimental studies have clearly demonstrated the impact of prematurity on nephron number [65]. In humans, the study by Crump et al. represents a milestone, showing an inverse relationship between gestational age and the risk of CKD from childhood to mid-adulthood [60]. Earlier evidence had already identified intrauterine growth restriction (IUGR), small for gestational age (SGA), and low birth weight (LBW) as risk factors for renal impairment, although these conditions often overlap [60,66]. For instance, Harer et al. reported that 25% of very low birth weight infants developed kidney impairment in early childhood [66]. Adolescents born preterm exhibit higher blood pressure and reduced renal function compared with those born at term [67]. These findings are consistent with incomplete nephrogenesis and reduced nephron endowment, which are associated with an increased lifelong risk of proteinuria, hypertension, and CKD [64,68,69,70,71]. A reduced nephron number leads to compensatory glomerular hyperfiltration, resulting in glomerular hypertrophy and increased susceptibility to kidney damage [65]. This mechanism has been demonstrated in specific populations, such as Australian Aboriginal groups, where low nephron number is linked to a higher CKD risk [72]. In preterm infants, nephrogenesis continues postnatally during the first 40 days of life [73]. However, this process occurs in an extrauterine environment and often results in the formation of structurally abnormal glomeruli. Autopsy studies have shown that preterm individuals frequently exhibit abnormal glomeruli in the outer renal cortex, where the most recently formed nephrons are located [64,74]. Consequently, postnatal nephrogenesis is generally suboptimal, leading to fewer and functionally impaired nephrons [75]. By adolescence, up to 50% of individuals born preterm may exhibit CKD-related abnormalities, including albuminuria, hypertension, or kidney hypoplasia [60]. Lower gestational age is also associated with reduced eGFR at 28 days of life, reflecting both glomerular and tubular immaturity [58,75]. Tubular dysfunction is characterized by increased sodium loss, although electrolyte balance tends to normalize earlier than proteinuria, which may persist longer [76]. Overall, individuals with reduced nephron endowment are more vulnerable to additional renal insults later in life [77].

### 4.2. Low Birth Weight, Intrauterine Growth Restriction and Small for Gestational Age

Low birth weight (LBW) is linked to hypertension and CKD, including progression to end-stage renal disease (ESRD) [78]. Evidence from large population-based studies supports this association. Vikse et al. demonstrated that LBW is associated with an increased risk of End-Stage Renal Disease (ESRD). In neonates with LBW, reduced nephron number has been associated with early signs of tubular dysfunction or injury, often accompanied by alterations in glomerular permeability [58]. LBW is considered a marker of reduced nephron endowment. However, its relationship with CKD is largely influenced by the underlying causes of LBW, particularly preterm birth and intrauterine growth restriction [78,79,80]. Placental perfusion abnormalities mostly cause it because of multiple factors, including multiple gestations, pre-eclampsia, chronic hypertension, diabetes, vascular disorders, thrombophilia, maternal smoking, and increased body weight [81,82,83,84].

Impaired placental function compromises fetal respiration, metabolism, and nutrient transfer [85]. This condition is often related to incomplete trophoblast invasion and inadequate remodeling of the maternal spiral arteries, exposing the fetus to a chronic environment of hypoxia and nutrient deprivation [86]. This phenomenon, at the cellular level, can be attributed to a reduction in the sphingolipid metabolite sphingosine-1-phosphate (S1P), which, through its signaling axis, plays a key role in placental angiogenesis, immune tolerance, and trophoblast proliferation and differentiation [87]. Nephrogenesis requires tightly regulated oxygen levels as it occurs within a physiologically hypoxic environment, and deviations in oxygen tension can have significant effects [88]. Pathological hypoxia alters hypoxia-inducible factor (HIF) activity, impairing ureteric bud branching, disrupting nephron progenitor differentiation, and affecting key developmental pathways, including Wnt/β-catenin and Notch signaling [89,90]. The severity and timing of hypoxia are critical: mild chronic hypoxia reduces nephron number, whereas more severe insults may lead to congenital anomalies of the kidney and urinary tract [91]. Consistent with these mechanisms, infants with IUGR show reduced nephron endowment, often with preserved glomerular volume. In contrast, children with low birth weight (<2.5 kg) exhibit both reduced nephron number and increased glomerular volume compared with those of normal birth weight [69,92]. Evidence from both human and animal studies supports the association between IUGR and alterations in renal structure, nephrogenesis, and vascular development [69,93]. During placental insufficiency, fetal hemodynamic adaptation prioritizes blood flow to vital organs such as the brain, heart, liver, and adrenal glands, at the expense of renal perfusion. This redistribution further impairs kidney development and exacerbates nephron deficit [94]. Since reduced nephron endowment is a recognized risk factor for hypertension and chronic kidney disease later in life, approximately 8% of pregnancies complicated by IUGR represent a population at significant long-term risk, highlighting the importance of placental insufficiency and pre-eclampsia in determining adult renal health [95,96].

Although the terms small for gestational age (SGA) and fetal growth restriction are often used interchangeably, SGA also includes constitutionally small but otherwise healthy fetuses [97]. While renal development is generally more severely affected in IUGR, children born with SGA still have a higher risk of renal impairment compared with the general population [86,98]. Basioti et al. reported that SGA infants show alterations in calcium and uric acid excretion at preschool age, suggesting early tubular dysfunction [98]. Furthermore, when combined with prematurity, SGA status has been associated with impaired glomerular function [99]. Reduced nephron endowment has also been described in this population [100]. Notably, Schmidt et al. demonstrated that weight for gestational age is an independent determinant of kidney size not only at birth but also during early childhood, with effects persisting at least up to 18 months of age. Their findings indicate that weight-for-gestational-age has a stronger influence on renal size than either birth weight or gestational age alone [101].

### 4.3. Drugs

Beyond reduced nephron endowment and renal immaturity, preterm infants are frequently exposed to additional renal insults, including nephrotoxic drugs and mechanical ventilation [70,77]. Acute kidney injury (AKI) is common in neonatal intensive care settings and acts as a “second hit” on renal development and long-term kidney health [102], representing an established precursor of CKD [103]. Neonatal AKI is defined as an increase in serum creatinine to 150–200% of baseline (stage 1), 200–300% (stage 2), or >300% or the need for dialysis (stage 3) [104]. It reflects an acute decline in renal function, with consequences for fluid and electrolyte homeostasis [105]. The most common mechanisms include renal hypoperfusion and exposure to nephrotoxic agents. Hypoperfusion is often secondary to cardiovascular instability and hypotension in conditions such as asphyxia, hemorrhage, sepsis, or patent ductus arteriosus (PDA). In these settings, renal autoregulatory mechanisms—mediated by prostaglandin-dependent afferent vasodilation and angiotensin-mediated arteriolar tone—may be impaired, leading to oliguria. In contrast, nephrotoxic injury, particularly drug-induced, may occur with preserved urine output, delaying recognition of renal dysfunction [106]. AKI itself increases the risk of subsequent CKD [107]. Extremely preterm infants who experience AKI after 40 days of life have been shown to have a lower nephron number than preterm infants without AKI [24]. AKI is most frequent in infants born before 29 weeks of gestation [108]. Pharmacological exposure is a major contributor to renal injury in this population. Preterm infants have higher total body water content than term infants [109], resulting in a larger volume of distribution for water-soluble antibiotics. At the same time, both glomerular filtration and tubular secretion are immature, reducing drug clearance [110].

### 4.4. Aminoglycosides

Gentamicin, combined with ampicillin, is recommended as first-line therapy for neonatal sepsis according to WHO guidelines [111]. Aminoglycosides are well known for their nephrotoxic effects, targeting proximal tubular epithelial cells, where they enter via endocytosis, inducing mitochondrial dysfunction and apoptosis [112,113,114]. Vancomycin is also associated with AKI, although at lower rates (1–9%), with a higher risk when combined with other nephrotoxic agents such as Nonsteroidal Anti-Inflammatory Drugs (NSAIDs) or aminoglycosides [115].

### 4.5. NSAIDs

NSAIDs, particularly indomethacin and ibuprofen, are widely used in neonatal intensive care for PDA closure [116]. Ibuprofen appears to be less nephrotoxic than indomethacin [117], while up to one-third of infants treated with indomethacin may develop AKI [118].

### 4.6. Mechanical Ventilation

Mechanical ventilation is an independent risk factor for AKI, as increased intrathoracic pressure can reduce venous return and impair renal perfusion [119]. In addition, antenatal glucocorticoids such as dexamethasone, administered to promote lung maturation, have been associated in animal models with reduced nephron endowment, impaired sodium handling, and increased blood pressure in offspring [120,121].

### 4.7. Atosiban

Tocolytic therapy with atosiban has also been linked experimentally to oligonephronia and renal vasodilation [122].

### 4.8. Syndromic Disorders: Down Syndrome and DiGeorge Syndrome

Down syndrome is the most common chromosomal disorder and is associated with a wide spectrum of clinical manifestations, including congenital heart disease, growth retardation, respiratory abnormalities, and hearing impairment. Despite these well-recognized comorbidities, current follow-up protocols do not routinely include structured nephrological monitoring [123,124]. Nevertheless, individuals with Down syndrome have a fivefold increased risk of nephro-urological abnormalities, as reported by Rossetti et al. [125]. Approximately one-quarter of patients present with renal hypoplasia and reduced eGFR compared with the general population [126]. Renal hypoplasia is commonly observed in this population and is associated with a limited renal functional reserve. This structural deficiency significantly increases the risk of CKD, with a notable decline in eGFR frequently emerging during adolescence [126]. In addition, these patients are more susceptible to autoimmune disorders, which may further contribute to renal involvement [127]. DiGeorge syndrome is primarily characterized by congenital heart defects, hypocalcemia, facial dysmorphisms, and neurocognitive impairment; however, approximately 30% of affected individuals also present congenital anomalies of the kidney and urinary tract (CAKUT) [128,129]. The severity of renal involvement and progression to CKD appears to depend on the specific genetic deletion, with the CRKL gene identified as a key determinant of renal impairment [130].

## 5. Postnatal Risk Factors

### 5.1. Early Life Nutrition and Its Impact on Renal and Cardiovascular Programming

Nutrition during infancy and early childhood plays a central role in growth and metabolic programming, with important implications for long-term renal health. A growing body of epidemiological and experimental evidence indicates that early dietary exposures can influence kidney development, nephron endowment, and later cardiovascular and renal outcomes [131,132,133]. Breastfeeding has consistently been associated with more favorable metabolic profiles, lower blood pressure in later life, and potentially improved renal outcomes. These benefits may be mediated by bioactive components of human milk, such as long-chain polyunsaturated fatty acids and their relatively low sodium content, which may support vascular development and blood pressure regulation across the life course [133,134]. In contrast, excessive protein intake during infancy, particularly from high-protein formulas, has been associated in some interventional studies with increased kidney volume and higher systolic blood pressure at school age. These findings suggest that early protein load may influence renal workload and long-term blood pressure trajectories [135,136]. However, large population-based cohort studies indicate that, after adjustment for confounding dietary and lifestyle factors such as sodium intake and overall diet quality, early protein intake may not be independently associated with kidney size or function at around 6 years of age, highlighting the complexity of nutritional effects on renal outcomes [136]. High sodium intake during early infancy and childhood has also been implicated in shaping long-term cardiovascular risk. Both observational and interventional data suggest that increased sodium exposure in early life may contribute to higher blood pressure and altered taste preferences, potentially affecting lifelong cardiovascular and renal regulation [133,134]. Overall, these findings support the concept that optimal early nutrition—including, when possible, breastfeeding, balanced macronutrient intake, and limited sodium exposure—represents a modifiable determinant of kidney health. Early dietary modulation during critical developmental windows appears to influence not only growth but also long-term renal and vascular programming [131,132,133].

### 5.2. Overweight and Obesity

Early life overweight has been associated with an increased risk of developing CKD after the age of 60 [137]. Liu et al. identified four studies evaluating childhood adiposity using body mass index (BMI) trajectories or overweight status, all of which reported significant associations with CKD in adulthood. Individuals with the highest BMI trajectories or greatest increases in BMI from childhood to adulthood showed the highest risk of CKD, suggesting a cumulative effect of elevated BMI on long-term renal outcomes [138]. Adipose tissue-derived cytokines contribute to both adaptive and maladaptive renal responses, including dysregulation of glomerular hyperfiltration. Several adipokines, such as leptin, adiponectin, vascular endothelial growth factor (VEGF), angiopoietins, and resistin, have been implicated in extracellular matrix accumulation and progressive renal fibrosis [139]. Beyond its metabolic role, adipose tissue functions as an endocrine organ that secretes pro-inflammatory mediators, including leptin, TNF-α, and interleukins, promoting renal inflammation, oxidative stress, and fibrotic remodeling, thereby accelerating CKD progression [140]. In addition, obesity is associated with activation of the RAAS, which promotes sodium retention and hypertension and exerts direct deleterious effects on renal structure and function [141].

### 5.3. Recurrent Urinary Tract Infections

Children frequently experience UTIs during early childhood, which may lead to renal scarring in up to 30% of cases [142]. Febrile UTIs can also represent an early manifestation of underlying structural abnormalities, such as vesicoureteral reflux [143]. Renal scarring is a recognized risk factor for the later development of hypertension and CKD, although this occurs only in a subset of patients [144]. However, evidence regarding a direct causal role of childhood UTIs in CKD development remains inconsistent. Population-based studies suggest that, in the absence of congenital or structural renal abnormalities, childhood UTIs alone are unlikely to be a major independent cause of CKD [145].

### 5.4. Exposure to Environmental Toxic Substances

Chronic high-level lead exposure is a well-recognized risk factor for CKD, with chronic tubulointerstitial nephropathy representing its main clinical manifestation [146,147]. However, even exposure at levels below those typically associated with overt lead nephropathy may act as a cofactor, increasing both the risk of CKD and its rate of progression [148]. In children, chronic low-level lead exposure has been associated with increased urinary excretion of biomarkers, including β2-microglobulin, prostaglandins, and tubular enzymes, indicating segment-specific nephron injury. Notably, these alterations have been observed at blood lead concentrations lower than those typically associated with renal effects in adults [149].

In addition, epidemiological data from large population-based studies confirm that low-level environmental lead exposure—at concentrations well below previously established safety thresholds—is associated with measurable reductions in glomerular filtration rate in adolescents [150]. This evidence reinforces the notion that there may be no safe level of lead exposure for the developing kidney and underscores the importance of revisiting current exposure limits, particularly in pediatric populations [150].

Similarly, cadmium exposure is nephrotoxic, with dose-dependent effects. Low-level exposure is primarily associated with proximal tubular dysfunction, whereas higher exposure levels can lead to glomerular injury. Cadmium accumulates in the kidney, particularly in proximal tubular cells, resulting in increased urinary excretion of biomarkers such as β2-microglobulin, retinol-binding protein, and N-acetyl-β-D-glycosaminidase, which are sensitive indicators of early tubular damage [151,152,153]. Emerging evidence also highlights the nephrotoxic potential of environmental mixtures; for instance, co-exposure to low levels of lead, cadmium, and arsenic during infancy has been shown to synergistically increase the risk of early signs of glomerular and tubular dysfunction, even when individual toxin levels remain below current safety thresholds [154]. Recent longitudinal evidence further strengthens the concept that early-life exposure to nephrotoxic metals may exert time-dependent effects on kidney development. Studies using innovative biomarkers, such as deciduous tooth analysis, have identified critical windows of susceptibility during prenatal life, particularly in the late second and third trimesters, during which exposure to metals such as lead, chromium, and lithium is associated with subsequent alterations in kidney function in preadolescence [154]. These findings suggest that even transient exposures during specific developmental periods may result in long-lasting changes in renal physiology, supporting the concept of developmental programming of kidney function [e1]. Notably, sex-specific differences have also been reported, with stronger associations observed in males, further highlighting the complexity of environmental influences on renal maturation [154].

### 5.5. Psychosocial Stress

Psychosocial stress has been associated with an increased risk of renal impairment in children, acting both as a trigger of acute functional changes and as a cofactor in long-term renal decline. In children with steroid-sensitive nephrotic syndrome, psychological stress has been shown to precede and predict the onset of proteinuria, suggesting a role in triggering disease relapses and negatively influencing renal outcomes [155]. Social and environmental determinants, including family dysfunction, socioeconomic disadvantages, and adverse childhood experiences, further amplify these risks and are associated with disparities in CKD prevalence and progression [156]. These mechanisms may be particularly relevant in the early development window. From a neurobiological perspective, the brain serves as the central organ orchestrating stress perception, adaptation, and recovery through a complex network involving the hippocampus, amygdala, and prefrontal cortex [157]. These structures regulate behavioral and physiological responses to stress via bidirectional communication with the autonomic nervous system, cardiovascular system, and immune pathways. While acute stress responses may be adaptive and support short-term homeostasis (allostasis), chronic exposure to stress can dysregulate these systems, resulting in a cumulative physiological burden, defined as allostatic load [157]. This chronic “wear-and-tear” effect has been associated with long-term impairment of multiple organ systems, including the kidneys. In addition, growing evidence from psychoneuroimmunology suggests that stress-induced activation of neuroendocrine pathways may directly influence immune function, promote systemic inflammation and alter cytokine profiles [158]. These immune-mediated mechanisms may further contribute to renal injury by enhancing inflammatory and oxidative pathways already implicated in CKD progression. The interplay between the central nervous system, endocrine responses, and immune regulation highlights the systemic nature of stress-related damage and its potential role in early renal programming [159]. Although most of the evidence linking stress to organ damage derives from cardiovascular research, important parallels can be drawn. Chronic psychological stress has been consistently associated with an increased risk of cardiovascular disease, while acute stress can act as a trigger for clinical events [160]. These findings support the concept that similar pathophysiological mechanisms—such as endothelial dysfunction, neurohormonal activation, and inflammation—may also contribute to renal vulnerability, particularly in developing individuals. Psychosocial stress should be considered not only a contextual factor but also a biologically relevant contributor to early-life renal risk. In vulnerable pediatric populations, chronic stress exposure may interact with other environmental and clinical factors, amplifying susceptibility to kidney dysfunction and potentially influencing long-term renal outcomes.

### 5.6. Gut Microbiota

The first 1000 days of life represent a critical window for establishing and maturing the gut microbiota, a process that plays an essential role in immune system development and metabolic homeostasis [161]. Increasing evidence supports a bidirectional gut–kidney axis, in which dysbiosis may contribute to long-term susceptibility to renal dysfunction [162,163]. Research by Li et al. indicate that the intestinal microbial landscape in CKD patients is defined by an enrichment of taxa such as Rothia and Helicobacter, alongside a depletion of Akkermansia and Clostridium IV. Notably, the authors highlighted the diagnostic potential of Akkermansia and Lactobacillus as biological markers for identifying the disease [164]. A healthy intestinal microbiota produces short-chain fatty acids (SCFAs), which have been shown to exert anti-inflammatory effects, regulate epithelial barrier integrity, and maintain intestinal permeability [165,166]. In addition, gut microbiota-derived uremic toxins, such as indoxyl sulfate and p-cresyl sulfate, have been thought to be involved in the progression of chronic kidney disease through activation of microinflammatory responses and fibrosis [162,167,168,169]. Overall, gut microbiota maturation during early infancy may represent an important determinant of long-term renal health, influencing immune development, metabolic regulation, and susceptibility to chronic kidney disease later in life [170,171].

## 6. Conclusions

**Figure 1:**
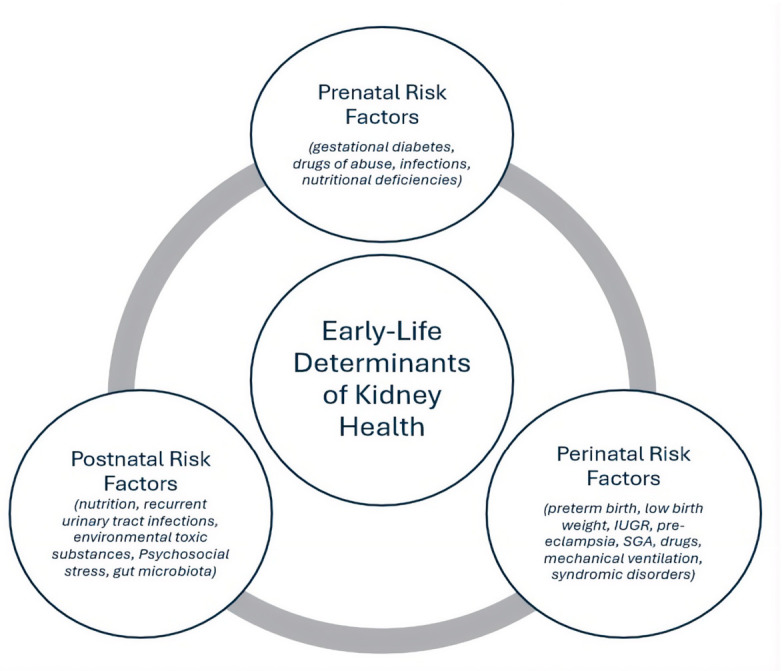
Early life risk factors across the lifespan contributing to chronic kidney disease susceptibility.

Preventing renal disease from fetal life through early childhood requires coordinated maternal, neonatal, and post-discharge strategies. Adequate maternal nutrition, including prevention of iron and zinc deficiencies, is essential to support fetal growth and early kidney development [44,49,172]. Low pre-pregnancy weight and inadequate gestational weight gain are associated with an increased risk of fetal growth restriction [173,174]. Screening for asymptomatic bacteriuria is also crucial to prevent urinary tract infections and reduce the risk of preterm birth [175]. In addition, maternal renal health, ranging from infection status to structural integrity, has important intergenerational effects on neonatal kidney development [176]. Prevention of placental insufficiency and intrauterine growth restriction should rely on optimization of maternal health before and during pregnancy, together with early identification of high-risk pregnancies [177]. Low-dose aspirin is widely supported for the prevention of placental complications [178]. In preterm infants, nephrogenesis continues for approximately 4–6 weeks after birth. Therefore, preventing kidney injury during this critical window is essential [179]. Clinical manifestations of renal dysfunction during this period may be delayed or nonspecific, including hypoperfusion, oliguria or anuria due to cortical necrosis, and electrolyte imbalance [180,181]. Renal protection in the neonatal intensive care unit requires careful monitoring of creatinine, electrolytes, fluid balance, weight, and acid–base status, together with the use of kidney-sparing medications [11,182]. Serum creatinine remains the most widely used biomarker of renal function, although it is influenced by age, muscle mass, renal maturation, and maternal levels in early life [183]. Cystatin C provides greater specificity, as it is independent of body composition and maternal contribution. More recently, urinary NGAL (neutrophil gelatinase-associated lipocalin) has emerged as a non-invasive marker of acute kidney injury, and together with urinary cathepsin B may help predict AKI after nephrotoxic exposure [75,183,184,185,186]. Outside the neonatal intensive care setting, non-invasive assessment of nephron endowment remains challenging. Kidney size and volume assessed by ultrasound are indirect indicators, as reduced renal dimensions often reflect low nephron number [187]. However, ultrasound cannot distinguish between physiological growth and compensatory hypertrophy, and therefore renal volume cannot be considered a direct surrogate of nephron number [188]. Similarly, estimated glomerular filtration rate has limitations in individual assessment, despite its usefulness in population studies [189]. Emerging technologies, such as transdermal sensors capable of detecting fluorescent filtration markers, may allow real-time estimation of kidney function and indirectly of nephron mass in the future [190]. Early surveillance is particularly important in vulnerable populations, including patients with Down syndrome and DiGeorge syndrome [191,192,193]. Attention should also be directed toward children exposed to environmental nephrotoxins such as lead and cadmium, through stricter regulation of contaminants in food, water, and consumer products and remediation of polluted areas [194,195]. In addition, pediatric follow-up should identify early trajectories toward obesity, using routine auxological monitoring to prevent long-term renal consequences [196,197,198]. Finally, urinary tract infections in infancy require careful attention, as renal scarring is more likely in this age group due to immune immaturity and a higher prevalence of underlying urinary tract anomalies. Early diagnosis and timely treatment are essential to reduce the risk of permanent renal damage [147,199]. Overall, the first 1000 days of life represent a unique developmental window in which maternal health, placental function, perinatal events, and early-life environmental exposures converge to determine lifelong nephron endowment and renal vulnerability. Early identification of at-risk pregnancies and infants, combined with prevention of renal insults during nephrogenesis, is essential to reduce the future burden of hypertension and chronic kidney disease (Figure 1).

Effective prevention across the first 1000 days requires a life-course approach that integrates maternal health optimization, early postnatal renal protection, biomarker-guided surveillance, and sustained follow-up of all at-risk infants to preserve nephron endowment and improve lifelong kidney health. Failure to identify and monitor these early-life determinants represents a missed opportunity for primary prevention of chronic kidney disease. Pediatricians occupy a strategic position in the structured clinical practice. As primary healthcare providers during the neonatal period and early childhood, they are uniquely positioned to identify individuals at increased risk of reduced nephron endowment or early renal injury and to implement longitudinal surveillance pathways to preserve kidney function across the lifespan. Pediatric nephrology outpatient follow-up could therefore be crucial among these at-risk pediatric groups.

## Data Availability

No new data were created or analyzed in this study. Data sharing is not applicable to this article.

## Abbreviations

AKI	Acute Kidney Injury
BMI	Body Mass Index
CAKUT	Congenital Anomalies of the Kidney and Urinary Tract
CI	Confidence Interval
CKD	Chronic Kidney Disease
CRKL	CRK Like Proto-Oncogene, Adaptor Protein
DOHaD	Developmental Origins of Health and Disease
eEGFR	Estimated Glomerular Filtration Rate
ESRD	End-Stage Renal Disease
EGFR	Glomerular Filtration Rate
GWAS	Genome-Wide Association Studies
HIFs	Hypoxia-Inducible Factors
IL	Interleukin
IUGR	Intrauterine Growth Restriction
KDIGO	Kidney Disease: Improving Global Outcomes
LBW	Low Birth Weight
NGAL	Neutrophil Gelatinase-Associated Lipocalin
NICU	Neonatal Intensive Care Unit
NO	Nitric Oxide
NSAIDs	Non-Steroidal Anti-Inflammatory Drugs
PDA	Patent Ductus Arteriosus
RAAS	Renin–Angiotensin–Aldosterone System
RR	Relative Risk
SBP	Systolic Blood Pressure
SGA	Small for Gestational Age
S1P	Sphingosine-1-Phosphate
SphK1	Sphingosine Kinase 1
S1PR	S1P Receptor
TNF-α	Tumor Necrosis Factor Alpha
UTI	Urinary Tract Infection
VEGF	Vascular Endothelial Growth Factor
WHO	World Health Organization
